# Harnessing anthocyanin-rich fruit: a visible reporter for tracing virus-induced gene silencing in pepper fruit

**DOI:** 10.1186/s13007-016-0151-5

**Published:** 2017-01-03

**Authors:** Jihyun Kim, Minkyu Park, Eun Soo Jeong, Je Min Lee, Doil Choi

**Affiliations:** 1 Department of Plant Science, Plant Genomics and Breeding Institute, College of Agriculture and Life Sciences, Seoul National University, 1 Gwanak-ro, Gwanak-gu, Seoul, 08826 Korea; 2 Department of Horticultural Science, Kyungpook National University, 80 Daehakro, Bukgu, Daegu, 41566 Korea; 3 Crop Biotechnology Institute/GreenBio Science and Technology, Seoul National University, Pyeongchang, Korea; 4 Department of Genetics, University of Georgia, Athens, GA 30602-7223 USA

**Keywords:** VIGS, Pepper, TRV, Reporter system, *An2*, Anthocyanin, Capsaicinoid

## Abstract

**Background:**

Virus-induced gene silencing (VIGS) has become a powerful tool for post-genomic technology in plant species. This is important, especially in select plants, such as the pepper plant, that are recalcitrant to *Agrobacterium*-mediated transformation. Although VIGS in plants has been widely employed as a powerful tool for functional genomics, scattering phenotypic effects by uneven gene silencing has been implemented in order to overcome challenges in experiments with fruit tissues.

**Results:**

We improved the VIGS system based on the tobacco rattle virus (TRV) containing the *An2* MYB transcription factor, which is the genetic determinant of purple colored- or anthocyanin-rich pepper. Silencing of endogenous *An2* in the anthocyanin-rich pepper with the modified TRV vector for ligation-independent cloning (LIC) lacked purple pigment in its leaves, flowers, and fruits. Infection with TRV–LIC containing a tandem construct of *An2* and *phytoene desaturase* (*PDS*) resulted in a typical photobleaching event in leaves without the purple pigment, whereas silencing of *PDS* led to the presence of photobleached and purple-colored leaves. Cosilencing of endogenous *An2* and *capsaicin synthase* in fruits resulted in decreased levels of capsaicin and dihydrocapsaicin as assessed by high performance liquid chromatography analysis coupled with the absence of the purple pigment in fruits.

**Conclusions:**

VIGS with tandem constructs harboring *An2* as a visible reporter in anthocyanin-rich pepper plants can facilitate the application of functional genomics in the study of metabolic pathways and fruit biology.

## Background

Over the last decade, more than 90 plant genomes and a large amount of transcriptome data have been available due to revolutionary advances in next generation sequencing technologies (https://en.wikipedia.org/wiki/List_of_sequenced_plant_genomes). Since the advent of the post-genomic era, functional genomics has been increasingly essential in order to identify genes of unknown function. In contrast to the innovative progress in generating sequence data, advances in functional studies of genes are many steps behind. Due to rapid straightforward methods, virus-induced gene silencing (VIGS) has become one of the most widely used tools in plant functional genomics [1, 2].

The pepper is a fleshy fruit-bearing plant and represents a nutritionally and economically important crop as a staple vegetable and food additive. Pepper consumption by humans has increased since it was determined that their fruits synthesize high levels of many health-promoting compounds including capsaicinoids, vitamin C, and carotenoids [3]. Therefore, breeding and biotechnology focused on enhancing the production of these metabolites are in demand although gene repertories regulating the pathways of these compounds are not fully understood. The pepper genome, however, can accelerate the identification of many genes potentially associated with important agronomic traits [4]. Pepper is closely related to the tomato plant, which is a model for fleshy fruit biology and biotechnology. Comparative genomics in Solanaceae accelerated the discovery of genes responsible for many agronomically important traits [4, 5]. Unlike tomato and other Solanaceae plants, pepper is recalcitrant to *Agrobacterium*-mediated-transformation; thus, VIGS has proven to be a powerful method for determining the function of unknown genes. The TRV system was widely employed for gene silencing in Solanaceae, including pepper plants [6–8]. These previous experiments were limited to vegetative tissues, which led to difficulties in the interpretation of the effects of virus and gene expression in the late stages of fruit development. Therefore, it is essential to develop molecular tools for the functional analysis of gene expression in pepper fruits responsible for biochemical processes of human health-promoting metabolites.

Although VIGS in plants has being widely employed as a powerful tool for functional genomics, scattering phenotypic effects driven by uneven gene silencing has become a problem that must be overcome, especially in fruit tissue. To ameliorate this limitation of VIGS, visual reporter systems were developed using GFP [9] and DEL-ROS [10] in GFP overexpressing and anthocyanin-enriched transgenic plants, respectively. Since silenced areas were visually traceable in these systems, dissection and sampling of tissues for subsequent metabolic analysis successfully identified gene functions impacting the metabolites of interest [9–11].

In this study, we improved the VIGS system to monitor gene silencing in pepper fruit. For this purpose, we employed the TRV-LIC VIGS system [12] for high-throughput cloning and used *An2* [13] as a reporter, which is the genetic determinant of purple pigmentation due to the accumulation of anthocyanin in pepper. We successfully examined the cosilencing effects of *PDS* and *capsaicin synthase* coupled with *An2*, and the subsequent phenotypic and metabolic changes in leaves and fruits, respectively. Our results advance reverse genetics methods for fruit traits and ultimately enhance the understanding of the molecular mechanisms of novel genes and regulators in fruit-specific metabolic pathways.

## Methods

### Plant materials and growing conditions

*Capsicum annuum* cv. NuMex Halloween (hereafter NMH), kindly provided by Prof. Byoung-Cheorl Kang (Seoul National University), was used in this study and maintained in growth chambers. Pungent NMH contained visible amounts of anthocyanin in leaf, stem, flower, and fruit. The plant height was approximately 30 cm. After germination on plates at 30 °C, the seedlings were transferred to plug trays at 25 °C with a 16/8 h light/dark photoperiod until cotyledons were fully expanded (approximately 2 weeks after germination). After agroinfiltration to the abaxial side of the cotyledons, pepper plants were incubated at 16 °C under dark conditions for 1 day. Four-week-post-infiltrated plants were transplanted to pots (130/115 mm) and grown at 20 °C with a 16/8 h light/dark photoperiod. The plants were fertilized by WUXAL according to the manufacturer’s instructions (WUXAL calcium, AGLUKON, Germany) once every 2 months.

### Plasmid construction

**Fig. 1:**
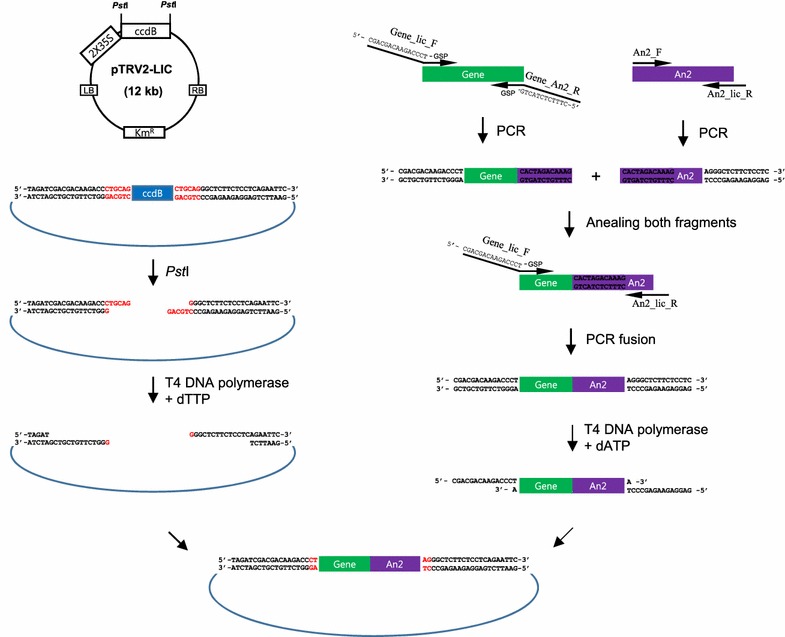
Cloning procedure using the *An2* reporter and the TRV2-LIC vector adapted from Dong et al. [12]. The TRV2-LIC vector was digested with *Pst*I and treated with T4 DNA polymerase and dTTP to generate sticky ends. The gene of interest was amplified by PCR using gene specific primers (GSP) and LIC (Gene_lic_F) and An2 adaptors (Gene_An2_R) using cDNA, and then treated with T4 DNA polymerase. *An2* was amplified using specific primers with an An2 adaptor (An2_F) and an LIC adaptor (An2_lic_R). The An2 adaptor sequence annealed to both the gene and *An2* fragments and a subsequent PCR using the LIC adaptor-attached primers fused the two fragments. The following PCR fragments were treated with T4 DNA polymerase and dATP to generate complementary sticky ends to anneal the ends of the linearized vector without DNA ligase. A mixture of both fragments was then transformed into *E. coli* DH5α

The TRV vectors, pTRV1 [14] and pTRV2-LIC [12], were kindly provided by Dr. Dinesh Kumar, at UC Davis. The ligation independent cloning (LIC) was conducted as described in Dong et al. [12] and Fig. 1. For gene cloning in pTRV2-LIC, the gene of interest (GOI) was amplified with primers: 5′-CGACGACAAGACCCT (LIC vector adaptor)-gene specific sequences-3′ and 5′-CTTTGTCTAGTG (*An2* adaptor)-gene specific sequences-3′ using SolgTM *Pfu*-X DNA polymerase (Solgent, Korea). *An2* was amplified with primers An2_F and An2_lic_R (Table 1). Fragments of *PDS* (CA03g36860, 173 bp), *CS* (CA02g19260, 181 bp), and *An2* (CA10g11650, 258 bp) were amplified from the pepper leaf, placenta, and pericarp cDNA, respectively. TRV2-GFP was previously used for VIGS as a control [15]. The insert size of each target gene and *An2* was 150–300 bp and the insert size of fused cDNAs was 400–600 bp in the TRV2-LIC vector. Using the amplified cDNAs of the target genes and *An2* as template, PCR was performed to fuse both cDNA fragments in the sense orientation. The PCR products were purified with DNA Clean and Concentrator™ (Zymo Research, USA). A total 100 ng of purified PCR product was treated with T4 DNA polymerase (New England Biolabs, USA) in 1× reaction buffer containing 10 mM dATP and dithiothreitol at 22 °C for 30 min followed by 20 min of inactivation of T4 DNA polymerase at 70 °C. The TRV2-LIC vector was digested by the restriction enzyme *Pst*I and treated with T4 DNA polymerase and dTTP instead of dATP. A total of 50 ng of PCR product and TRV2-LIC vector were mixed and incubated at 65 °C for 1 min and then 22 °C for 10 min. The mixture was transformed into *E. coli* DH10B or DH5α competent cells. Transformants were selected by PCR using primers for sequencing (Table 1) and confirmed by DNA sequencing. The plasmids from the transformants were introduced into *Agrobacterium tumefaciens* strain GV3101 using the freeze–thaw method [16].

**Table 1:** List of primer sequences used in this study

Primer	Sequence (5′–3′)
*VIGS construct*	
CS_lic_F	CGACGACAAGACCCTGAGAAGGGAAACTGCCATTTGA
CS_An2_R	CTTTGTCTAGTG CCTTGCCCAGCTTTGTAATCTT
GFP_lic_F	CGACGACAAGACCCTCACGGCAGACAAACAAAAGA
GFP_An2_R	CTTTGTCTAGTG AAAGGGCAGATTGTGTGGAC
An2_F	CACTAGACAAAGACGAACGCGAC
An2_lic_R	GAGGAGAAGAGCCCTCAGAAAAGTCATCCCAACCATCAC
PDS_lic_F	CGACGACAAGACCCTCTTGCAAAGATCCCCTGTAG
PDS_An2_R	CTTTGTCTAGTGCACTTGTTTCTGCCAACTTC
*Sequencing*	
TRV2_seq_F	CTGTTTGAGGGAAAAGTAG
TRV2_seq_R	CAAAAGACTTACCGATCAATC
*qRT-PCR*	
CS_F	TTCCCATATAGCCCACTTGC
CS_R	ACTACAAGCAAATTACCACCTTC
PDS_F	AGCAAAGCAAAAATATTGAAGTA
PDS_R	GCTTTCCTGATAAGACAGC
An2_F	GGAGAAGGAAAGTGGCATCTTGT
An2_R	CACCTCTCTTTATATGCGGCCTT
CaActin_F	ATGGCAGATGAAGATATTCAAC
CaActin_R	ACTAGGAAAAACAGCCCTTGGT

### Agroinfiltration

*Agrobacterium tumefaciens* strain GV3101 carrying pTRV1, pTRV2::*GFP*, pTRV2-LIC::*GFP*::*An2*, pTRV2-LIC::*PDS*::*An2*, pTRV2-LIC::*CS*::*An2*, and was grown overnight at 28 °C in 10 mL YEP medium containing rifampicin (50 µg/mL) and kanamycin (50 µg/mL) as described [6]. The transformed *Agrobacterium* was harvested by centrifugation at 13,000×*g* for 15 min at 20 °C, and resuspended in 10 mM MES, 10 mM MgCl_2_, and 200 µM acetosyringone to a final OD_600_ nm of 0.7. Cell suspensions were incubated at room temperature with inverting for 4 h. *Agrobacterium* cultures containing pTRV1 and pTRV2-LIC carrying any GOI were mixed at a 1:1 ratio and infiltrated into the abaxial side of the both cotyledons. Leaves from 4-week-old plants and placenta from fruits from plants 30 days post-anthesis (DPA) were harvested and immediately frozen in liquid nitrogen for qRT-PCR and HPLC. Due to a high frequency of fruit abscission, at least five silenced plants per each construct were grown at 20 °C until harvest.

### RNA isolation and quantitative RT-PCR analysis

The total RNA from 100 mg of tissue was extracted using the TRIzol^®^ reagent (Invitrogen, USA). Total RNA (5 µg) was reverse-transcribed with Oligo (dT) primers and Superscript II (Invitrogen), according to the manufacturer’s instructions. Subsequently, qRT-PCR was conducted to analyze gene expression level using the SYBR Green PCR master mix (Invitrogen, USA) and gene-specific primers (Table 1) in the Rotor-Gene 6000 apparatus (QIAGEN, USA), according to the manufacturer’s instructions. All statistical analyses were conducted as described in the manufacturer’s protocol. To normalize the expression levels, the transcript level of *CaActin* (CA00g80270) was used as a control. Duplicates from 3 biological replicates were used in the qRT-PCR analysis.

### Capsaicinoid extraction and HPLC analysis

The placental tissue of each fruit at 30 days-post anthesis was isolated and immediately frozen in liquid nitrogen. For sampling in *An2*-silenced plants, purple pigment-depleted placenta was carefully collected. Previous method of capsaicinoid extraction [17] was modified for frozen tissues. Approximately 100 mg of frozen powder from pooled samples of two fruits was extracted with 1.5 mL of an ethyl acetate and acetone mixture (6:4) using TissueLyser II (QIAGEN, USA) at room temperature for 10 min and incubated with shaking at 37 °C for 1 h. After centrifugation at 12,000×*g* for 5 min at room temperature, 600 µL of the supernatant was transferred to a new tube and evaporated in an Automatic Environmental SpeedVac System AES1010 (Operon, Korea). The extract was dissolved in 500 µL methanol and filtered using an Acrodisc^®^ LC 13-mm syringe filter with a 0.2-µm PVDF membrane (Sigma-Aldrich, USA). Capsaicinoid analysis was performed using the UltiMate^®^ 3000 HPLC (Dionex, USA) including the Inno C-18 column (4.6 mm × 150 mm, YoungJin Biochrom, Korea). A UV detector was operated at 280 nm and the data acquisition was performed with Chromeleon software. Separation of capsaicinoids was achieved under 75% MeOH at a flow rate of 1 mL/min. Each 10 µL aliquot was analyzed with HPLC. The HPLC analyses were performed at NICEM, Seoul National University. Capsaicin and dihydrocapsaicin used as standard compounds were purchased from Sigma-Aldrich (M2028 and M1022, respectively) [17].

## Results

### Improvement of VIGS in the pepper plant using a TRV2-LIC system and *An2*

**Fig. 2:**
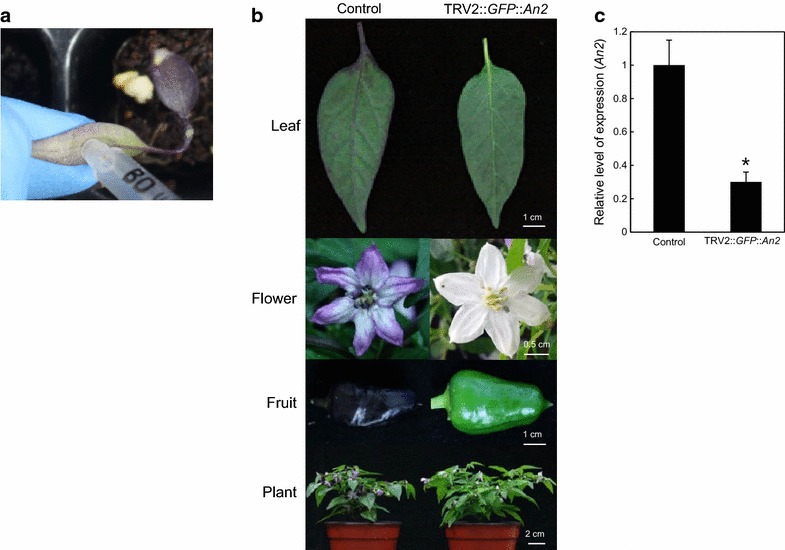
Anthocyanin-mediated visualization of VIGS in peppers using TRV2-LIC and *An2* as a reporter. **a** Agroinfiltration to NMH cotyledons. **b** Results from different tissues of TRV2-*GFP*-infiltrated pepper (control, *left*) and TRV2::*GFP*::*An2* infiltrated pepper (*right*). **c** Expression analysis of *An2* by qRT-PCR in 30 DPA fruits. Data indicate relative expression level compared to control. Three biological replicates of qRT-PCRs were performed. Data are shown as mean ± SE. Statistically significant differences, determined by Student’s t test, are indicated by an *asterisk* (*P* ≤ 0.05)

The purpose of this study was to develop efficient gene silencing in pepper fruit based on the TRV2-LIC system [12]. The TRV2-LIC system utilizes the exonuclease activity of T4 DNA polymerase to generate sticky ends in both the insert and the vector (Fig. 1). Then, *ccdB* in the vector was used to efficiently select putative recombinant colonies. Therefore, the TRV2-LIC system enabled us to clone any gene of interest (GOI) in a high-throughput manner. Although TRV systems were successfully used previously in Solanaceae, VIGS in pepper fruits has not been fully investigated. To improve silencing efficiency and uniformity in fruit, we used *An2* as a reporter gene in the TRV2-LIC construction to visualize the sector subject to silencing in the purple pepper. The *A* locus controls anthocyanin accumulation in various tissues of the pepper plant [18] and the R2R3-MYB transcription factor, *An2*, has been identified as the candidate gene of the *A* locus [13]. The expression of *An2* was detected in all stages of fruit development and also in both the flower and leaf, resulting in anthocyanin accumulation. To verify the function of *An2* and its possible use as a reporter for VIGS, a gene specific fragment of *An2* (CA10g11650, 258 bp) was incorporated into the TRV2-LIC vector (Figs. 1, 2b).

### Silencing and its assessment of endogenous *An2* in different tissues

Agrobacterium carrying TRV2::*GFP* as a control and TRV2::*GFP*::*An2* with TRV1 were infiltrated in the cotyledons of 2-week-old seedlings of NMH to extend the silencing response to meristematic tissues (Fig. 2a). *GFP* was used for control of gene fragment and the *GFP* gene fragment is not existed in the pepper genome. Therefore, *GFP* in the TRV2-LIC would not affect any gene expression or phenotype. The infiltrated plants were grown at 20 °C to maintain silencing for a long period and enhance silencing efficiency. NMH, dwarf, and ornamental pepper cultivars, were characterized by purple leaf pigmentation, flowers, and immature fruits, and harbored orange colored ripe fruits. Fruits were oriented upright, were bullet shaped, and pungent (http://www.chilepepperinstitute.org/cart/product/100/numex_halloween/). Figure 2b demonstrates the phenotypes of the control and *An2*-silenced peppers in different tissues. *An2* silencing using TRV2-LIC systems clearly resulted in anthocyanin deficiency in leaves, flowers, and fruits. In flowers of *An2*-silenced plants, the absence of the purple pigment was also observed in the stigma and stamen. To examine whether the fruit phenotypes observed in the infiltrated peppers were associated with the down-regulation of *An2*, qRT-PCR was conducted to compare the gene expression between purple and green fruits. The level of *An2* expression was remarkably decreased in TRV2::*GFP*::*An2* compared to the control (Fig. 2c), and the impact of *An2* silencing was negligible compared to the control. Therefore, *An2* was chosen as a reporter in this VIGS system for NMH by monitoring anthocyanin accumulation. These results also indicate that *An2* is the genetic determinant of the *A* locus [13].

### Validating the cosilencing method in the tandem construct using *PDS* and *An2*

**Fig. 3:**
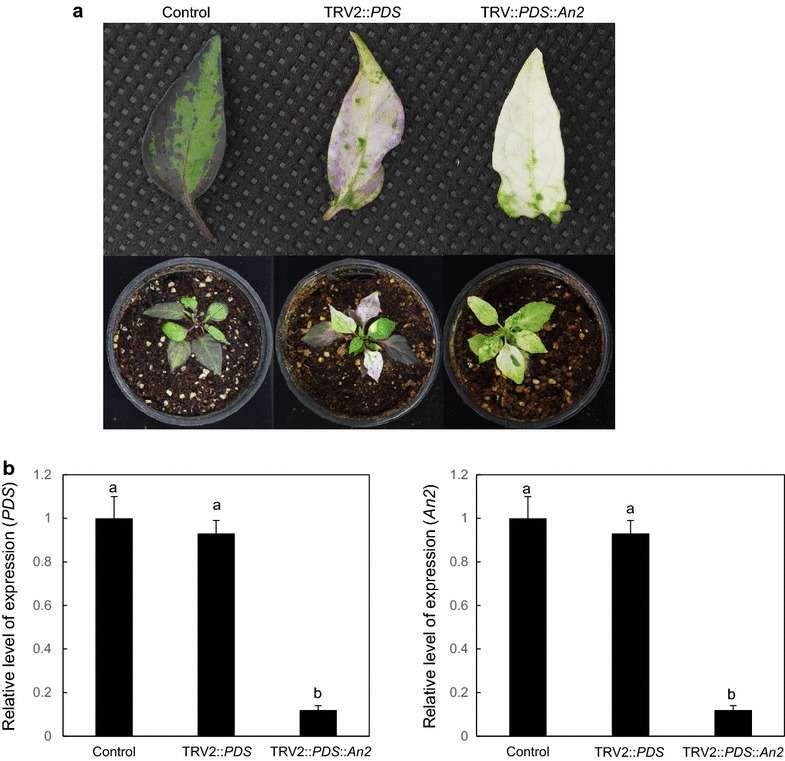
Co-silencing of *PDS* and *An2* in NMH leaf. **a** Two week-old plants were agroinfiltrated to the abaxial side of the cotyledons with *Agrobacterium* cultures of pTRV1/pTRV2::*GFP* (control), pTRV1/pTRV2::*PDS*, and pTRV1/pTRV2::*PDS*::*An2*. *PDS*/*An2*-silenced plants resulted in photobleaching and a lack of purple pigment, whereas *PDS*-silenced plants were characterized by purple pigmentation under white leaves 5 weeks post-infiltration. **b** In the qRT-PCR analysis, *An2* expression level in the leaves of *PDS*/*An2*-silenced plants was generally lower than those in the control or *PDS*-silenced plants. However, *PDS* expression level remarkably decreased in *PDS*/*An2*- and *PDS*-silenced plants compared to the control. Data indicate relative expression compared to the control. All the qRT-PCRs were performed using three biological replicates. Data are shown as mean ± SE. The *different letters* indicate significant difference (*P* ≤ 0.05) by Duncan test

To validate the cosilencing effect of *An2* and GOI, the prevention of purple pigmentation by *An2* silencing and subsequent phenotypic analysis by GOI silencing were investigated. As a proof of concept, we employed *PDS* to verify that cosilencing occurred with *An2* in NMH. A gene specific fragment of *PDS* (CA03g36860, 173 bp) was tandemly fused with *An2* in the TRV2-LIC system and this construct was then agroinfiltrated in the cotyledons of NMH (Fig. 1). Subsequently, *PDS* and *An2* mRNA levels were measured using qRT-PCR in 4-week-old leaves. Silencing of *PDS* resulted in photobleaching of NMH leaves and did not prevent anthocyanin pigmentation. However, cosilencing of *PDS* and *An2* was shown to cause photobleaching coupled with a lack of anthocyanin in leaves of NMH (Fig. 3). There were no significant differences in the levels of *PDS* mRNA between TRV2::*PDS* and TRV2::*PDS*::*An2* leaves, suggesting silencing of *An2* did not affect *PDS* expression. The expression level of *PDS* significantly decreased in both TRV2::*PDS* and TRV2::*PDS*::*An2* leaves. However, *An2* expression notably decreased only in TRV2::*PDS*::*An2* leaves.

### Validating cosilencing in fruits using *capsaicin synthase* and *An2*

Capsaicinoids are specialized metabolic determinants of pungency and natural vanilloid, which are only found in pepper species. Capsaicin synthase (CS), a homologue of acyltransferase, is the genetic factor of the *C* or *Pun1* locus controlling pungency [19]. Capsaicin synthase catalyzes the last step of the pathway by condensing vanillylamine to 8-methyl-6-nonenoyl-CoA [20]. Capsaicinoids are mostly synthesized in placenta tissue and capsaicin synthases are only expressed during pepper placenta development [19].

**Fig. 4:**
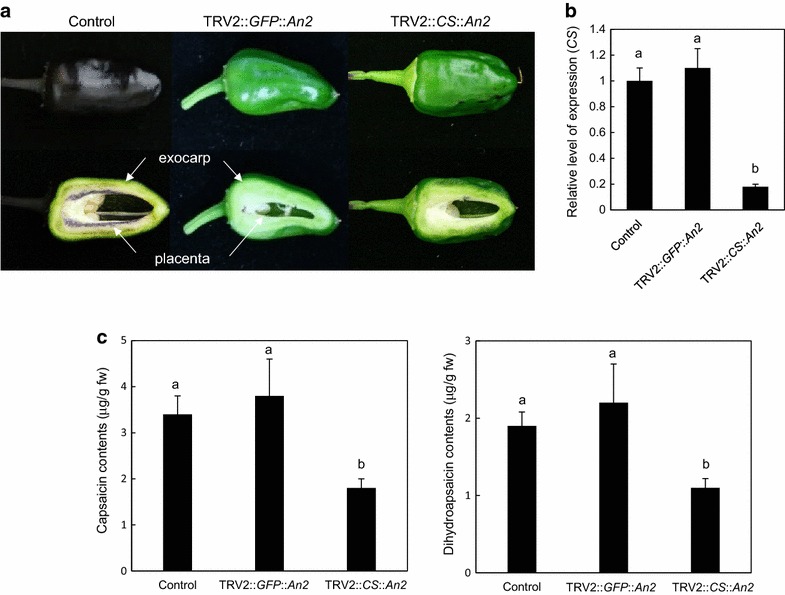
Cosilencing of *capsaicin synthase* (*CS*) and *An2*. **a** Anthocyanin-mediated visualization of cosilencing of *CS* and *An2* in pepper pericarp and placenta (30 DPA). Silencing of *An2* resulted in the depletion of anthocyanin pigmentation in the exocarp and placenta of TRV2::*GFP*::*An2* and TRV2::*CS*::*An2*. **b** Gene expression analysis of *CS* in the cosilenced fruits (placenta) compared to the control and *An2*-silenced fruits by qRT-PCR. The expression levels of *CS* were significantly lower in TRV2::*CS*::*An2* than in the control and TRV2::*GFP*::*An2*. **c** Capsaicinoid analysis in the cosilenced fruits compared to control and *An2*-silenced fruits by HPLC (n = 6). Cosilencing of *An2* and *CS* in the placenta resulted in decreased levels of capsaicin and dihydrocapsaicin. *Asterisks* indicate significant differences (*P* ≤ 0.05) between TRV2::*GFP*::*An2* and TRV2::*CS*::*An2*. Data are shown as mean ± SE. The *different letters* indicate significant difference (*P* ≤ 0.05) by Duncan test

To validate the cosilencing effect of GOI with *An2* in fruits and its potential use as a tool for metabolic genetics, a partial cDNA of *CS* (CA02g19260, 181 bp) was tandemly fused with *An2* in the TRV2-LIC system and this construct was agroinfiltrated as described above. To maintain and allow for the VIGS signal to reach fruits, the agroinfiltrated peppers were grown at 20 °C until harvest. Due to the spatiotemporal regulation of capsaicinoid biosynthesis and *CS* expression, placenta tissues of at least six different 30 DPA fruits were collected for further quantification of capsaicinoid and *CS* transcripts. The visual phenotypes of representative fruits, resulting quantification of *CS* transcripts as well as the capsaicinoids are shown in Fig. 3. In the control, anthocyanin mainly accumulated in the exocarp (not the endocarp) and was clearly visible in placenta tissues (Fig. 4a). Silencing of *An2* resulted in the loss of anthocyanin pigmentation in the exocarp and placenta of TRV2::*GFP*::*An2* and TRV2::*CS*::*An2*. To assess the cosilencing effects of *CS* and *An2* in the placenta, the anthocyanin-depleted placenta (30 DPA) was carefully dissected and used for further analysis with qRT-PCR and HPLC. There were no significant differences in the levels of *CS* mRNA between the control (TRV2::*GFP*) and TRV2::*GFP*::*An2* leaves, suggesting that silencing of *An2* did not affect *CS* expression. The expression levels of *CS* were significantly lower in TRV2::*CS*::*An2* than in the control or TRV2::*GFP*::*An2* (Fig. 4b). To address whether *CS* silencing affected capsaicinoid accumulation in the placenta, capsaicinoids were quantified in the same tissues used for qRT-PCR. The HPLC method was able to identify biosynthesis of capsaicin and dihydrocapsaicin, which are major the capsaicinoids in pepper fruits. Thus, *An2* silencing did not affect capsaicinoid content. However, *CS*-silenced plants showed a significant 50% reduction of capsaicin and dihydrocapsacin, as expected, in 30 DPA-placenta (almost 50%) compared to the control and TRV::*GFP*::*An2* (Fig. 4c). Any other phenotypic changes caused by cosilencing *CS* and *An2* was not observed compared to the *An2*-silenced plant, suggesting that these systems are feasible methods to use in functional plant genomics. Metabolic phenotypes of the silenced fruits were in agreement with enzyme function. The anthocyanin accumulation or depletion in silenced placenta tissues had no significant effect on capsaicinoid biosynthesis or expression of *CS*.

## Discussion

A reporter gene is required in VIGS to visualize the silenced region and monitor the silencing efficiency. *PDS* has been widely used as a reporter of choice [21]. Since *PDS* silencing decreased chlorophyll and carotenoid biosynthesis resulting reduced photosynthetic activity, *PDS*-silenced plant showed growth defect [22]. *PDS* silencing also caused carotenoid-deficient fruits and affected other metabolisms beyond carotenoid in tomato fruits [10, 23]. Thus, use of *PDS* as a reporter expect to show poor fruit set and disturb important metabolisms in pepper fruits. Unlike *PDS*, *An2* silencing did not affect other phenotype except anthocyanin pigmentation. Therefore, we choose *An2* as a reporter in this study.

Despite the advantages and utility of VIGS in plant functional genomics studies [1, 2], the uneven distribution of silencing in target tissues is a major limitation. Use of a visible reporter would lead to the efficient sampling of silenced tissues from nonsilenced tissues, therefore increasing the sensitivity of subsequent analysis. Several studies have successfully overcome this limitation using transgenic plants of DEL-ROS [1, 10, 11] and GFP [9]. Using the transgenes as visible reporters, cosilencing with the reporter and GOI enables us to identify the precise region where silencing occurs. Ectopic expression of transcription factors, *DEL* and *ROS1* using a ripe-fruit specific promoter resulted in the up-regulation of the anthocyanin biosynthetic genes, leading to anthocyanin accumulation in ripening tomato fruits. Cosilencing the reporter genes with GOI in the transgenic plants facilitated visualization of the silenced region. Fluorescence of the GFP-silenced region in GFP-overproducing plants was clearly decreased after exposure to UV. Therefore, target tissues can be easily collected and the effect of silencing can be more easily identified in systems with higher phenotypic variation compared to the non-collective method. However, these methods are still limited in plant species, which are recalcitrant to *Agrobacterium*-mediated transformation. In order to extend this concept to most the majority of plant species, we established the endogenous reporter system, *An2*, under natural variation of anthocyanin-rich pepper. Although we intended to develop this system for fruit genetics, the reporter in NMH can easily be used to monitor the silenced sector of genes in leaves and flowers and facilitate collection of tissues without further expression analysis of GOI, as we revealed that it was possible to cosilence *PDS* and *An2* (Fig. 3). Therefore, our results will be helpful to study genes controlling diverse biological phenomena. Previous studies on *CS* knockdown by VIGS showed reduced capsaicinoid content in fruits, which is in agreement with the results of our study (Fig. 4) [19, 24], clearly indicating that *CS* is responsible for capsaicinoid synthesis. *CS*, originally referred to as *Pun1*, is believed to catalyze the last step of capsaicinoid biosynthesis although there is no direct evidence that Pun1 has capsaicin synthase activity. Recently, using a protoplast-based assay for *de novo* capsaicin synthesis and antibodies of the anti-Pun1, which are antagonists of endogenous Pun1 activity, the *Pun1* gene and its product were proven to be involved in capsaicin synthesis [24]. These data together with our results reveal that CS or Pun1 primarily controls the final step in capsaicinoid biosynthesis.

VIGS using TRV systems efficiently functioned in vegetative pepper tissues [6]. However, the silencing signal is not well transmitted throughout the whole plant, particularly in reproductive tissues. Silencing of *PDS* was previously maintained in flowers and tomato fruits and enhanced by low temperature and low humidity [25]. In our study, low temperature was a critical requirement of VIGS in fruits, although fruits were often characterized by evidence of abscission before harvest (data not shown). Further improvement using different environmental conditions and diverse pepper cultivars should be thoroughly examined. In addition, *An2* as a transcription factor, should not dramatically affect the developmental or biochemical regulation underlying the function under study, for example, in cases when the GOI is a transcription factor or other master regulator.

## Conclusions

Here, we present the utility of the gene silencing approach, which uses an endogenous reporter, *An2*, in anthocyanin-rich peppers, both by down-regulating *PDS* in leaves and *CS* in fruits, ultimately demonstrating the feasibility of this system in metabolic genetics in pepper fruits. Our results demonstrate the power of this tool for addressing the roles of regulatory genes in capsaicinoid biosynthesis and secondary metabolism. The application of this system to examine candidate regulatory genes will be useful and facilitate confirmation of gene functions identified in the model species: tomato and *Arabidopsis*.

## Abbreviations

VIGS: virus-induced gene silencing
TRV: tobacco rattle virus
GOI: gene of interest
LIC: ligation independent cloning
PDS: phytoene desaturase
CS: capsaicin synthase
HPLC: high performance liquid chromatography
DPA: days post-anthesis
